# ALKBH5-mediated m^6^A demethylation of KCNK15-AS1 inhibits pancreatic cancer progression via regulating KCNK15 and PTEN/AKT signaling

**DOI:** 10.1038/s41419-021-04401-4

**Published:** 2021-12-01

**Authors:** Yuan He, HongQin Yue, Ying Cheng, Zhilong Ding, Zhen Xu, Chunyang Lv, Zheng Wang, Jing Wang, Chenglong Yin, Huihui Hao, Chuang Chen

**Affiliations:** 1 Department of General Surgery, Heping Hospital, Changzhi Medical College, Changzhi, Shanxi 046000 China; 2 Department of Hepatopancreatobiliary Surgery, Huai’an Hospital Affiliated to Xuzhou Medical University (Second People’s Hospital of Huai’an City), Huai’an, Jiangsu 223002 China; 3 Department of Gastroenterology, the Yancheng School of Clinical Medicine of Nanjing Medical University, Yancheng, Jiangsu 224000 China; 4 Department of Hemodialysis Room, Huai’an Hospital Affiliated to Xuzhou Medical University (Second People’s Hospital of Huai’an City), Huai’an, Jiangsu 223002 China; 5 Department of Pharmacology, Jiangsu College of Nursing, Huai’an, Jiangsu 223002 China

**Keywords:** Cancer, Diseases

## Abstract

Long noncoding RNAs (lncRNAs) are regarded as crucial regulators in tumor progression. Potassium two pore domain channel subfamily K member 15 and WISP2 antisense RNA 1 (KCNK15-AS1) has been confirmed to inhibit the migration and invasion of pancreatic cancer (PC) cells. However, its downstream mechanism and effect on other cellular functions in PC remain unknown. This study probed the function and potential mechanism of KCNK15-AS1 in PC cell growth. RT-qPCR and western blot were employed to measure gene expression in PC cells. ISH was applied to analyze KCNK15-AS1 expression in PC tissues. Functional assays were utilized to evaluate PC cell proliferation, apoptosis, migration and EMT. Mechanical experiments were adopted to detect gene interaction in PC cells. The obtained data indicated that KCNK15-AS1 was down-regulated in PC cells and tissues. Overexpressing KCNK15-AS1 hindered cell proliferation, migration and EMT while facilitated cell apoptosis in PC. Mechanically, alkylation repair homolog protein 5 (ALKBH5) was verified to induce m^6^A demethylation of KCNK15-AS1 to mediate KCNK15-AS1 up-regulation. KCNK15-AS1 combined with KCNK15 5’UTR to inhibit KCNK15 translation. Moreover, KCNK15-AS1 recruited MDM2 proto-oncogene (MDM2) to promote RE1 silencing transcription factor (REST) ubiquitination, thus transcriptionally upregulating phosphatase and tensin homolog (PTEN) to inactivate AKT pathway. In conclusion, our study first confirmed that KCNK15-AS1 hinders PC cell growth by regulating KCNK15 and PTEN, suggesting KCNK15-AS1 as a potential biomarker of PC.

## Introduction

Pancreatic cancer (PC), a prevalent malignancy, is one of the top four fatal cancers worldwide [1]. The overall 5-year survival rate of PC patients is low, nearly 5% [2]. Surgical resection and chemotherapy are main treatment options for PC, but neither has satisfactory efficacy [3, 4]. Since the molecular mechanism of PC progression is poorly understood [5], more efforts are needed in this field.

Recently, whole genome and transcriptome sequencing technologies have gradually revealed the importance of non-coding RNAs (ncRNAs) [6]. Long non-coding RNAs (lncRNAs) are a class of ncRNAs with over 200 nucleotides in length and without protein-coding potentials [7]. As reported, lncRNAs participate in various cellular processes, such as proliferation, differentiation, and apoptosis [8], thus working as oncogenes or tumor suppressors in diverse cancers including PC [9]. For example, ZEB2-AS1 accelerates PC cell growth via miR-204/HMGB1 axis [10]. GAS5 represses PC cell metastasis by sponging miR-221 to affect SOCS3 [11]. LncRNA potassium two pore domain channel subfamily K member 15 and WISP2 antisense RNA 1 (KCNK15-AS1) has been reported to inhibit gastric cancer progression via regulating DNMT1-MAPK and HDAC1-AKT axes [12]. Importantly, a previous study has revealed that KCNK15-AS1 is downregulated in PC and negatively regulates PC metastasis [13]. Nevertheless, the impact and related mechanism of KCNK15-AS1 on PC tumorigenesis remain unknown.

As one of the most common RNA modification modes, N^6^-methyladenosine (m^6^A) modification is an invertible procedure regulated by METTL3, METTL14, WTAP, FTO, and ALKBH5 [14]. This modification can affect mRNA transportation, shearing, translation as well as post-translational processing [15]. Presently, reports have certified that ALKBH5-mediated m^6^A demethylation of target RNAs is relevant with the progression of many cancers, including PC [16]. For instance, ALKBH5 impedes PC tumorigenesis by inhibiting Wnt signaling [17]. In our study, we investigated the correlation between m^6^A modification and KCNK15-AS1 expression in PC.

AKT is a serine/threonine kinase vital for PI3K signaling [18], and PI3K/AKT pathway regulates many growth factors [19]. Upon PI3K stimulation, AKT is phosphorylated and activated, therefore promoting cell survival, proliferation, and migration [20]. Phosphatase and tensin homolog (PTEN) is a lipid phosphatase that blocks AKT phosphorylation to inactivate this pathway [21]. In this regard, PTEN is recognized as a classic tumor suppressor [22]. The involvement of PTEN/AKT pathway has been widely reported in many cancers, including PC [23]. For example, CBX7 impedes cell proliferation and migration via inactivating PTEN/AKT signaling in PC [24].

Here, we aimed to probe the role and mechanism of KCNK15-AS1 in regulating PC cell growth. Our findings might support KCNK15-AS1 as a potential biological marker for PC.

## Materials and methods

### Cell culture

American Type Culture Collection (ATCC; Manassas, VA, USA) offered four human PC cells including PANC-1, CFPAC-1, MIA-PaCa-2, and BxPC-3. Human normal pancreatic ductal epithelial cells HPDE6-C7 were obtained from FengHui biology (Hunan, China). Dulbecco’s Modified Eagle’s Medium was used for the cultivation of PANC-1 and MIA-PaCa-2 cells. Iscove’s Modified Dulbecco’s Medium was used for culturing PANC-1 cells. RPMI-1640 Medium was employed for cultivating BxPC-3 cells. McCoy’s 5a medium was adopted to culture HPDE6-C7 cells. All cell media were added with 10% fetal bovine serum and cultured in 5% CO_2_ at 37 °C.

### In situ hybridization (ISH)

PC tissues and adjacent normal tissues were obtained from Huai’an Hospital Affiliated to Xuzhou Medical University (Second People’s Hospital of Huai’an City). Patients had signed the informed consent before this study. This study had been approved by the Ethics Committee of Huai’an Hospital Affiliated to Xuzhou Medical University (Second People’s Hospital of Huai’an City). The investigator was blind to the group allocation during the experiment.

Slides were deparaffinized and rehydrated, and then digested with proteinase K. Subsequently, digoxin-labeled KCNK15-AS1 probe was incubated in hybridization buffer. After washing and blocking, the samples were incubated with antibodies including anti-digoxin and anti-horseradish peroxidase. After washing and hematoxylin staining, DAB was utilized for visualization and the pictures were obtained using a microscopy. Experiments were repeated three times.

### Quantitative real-time polymerase chain reaction (RT-qPCR)

Trizol reagent (Invitrogen, USA) was adopted for total RNA extraction, followed by reverse transcription. QPCR was carried out by SYBR Premix ExTaq kit (Takara, Japan) based on the 2^−ΔΔCt^ method. The expression of RNAs was normalized to GAPDH. Each sample was tested in triplicate.

### Cell transfection

For gene overexpression, cells were transfected with empty vector or overexpressing vector of KCNK15-AS1, ALKBH5, KCNK15, or REST (GenePharma Company). For gene knockdown, sh/Ctrl, ALKBH5 shRNAs (sh/ALKBH5#1/2/3), PTEN shRNAs (sh/PTEN#1/2), and MDM2 shRNA (sh/MDM2) were acquired from GenePharma (Shanghai, China). All transfection was implemented via Lipofectamine 3000 (Invitrogen, USA) and transfection efficiency was verified using RT-qPCR.

### 5-Ethynyl-2′-deoxyuridine (EdU) assay

Cell proliferation was examined using an EdU Kit (Ribobio) according to supplier’s advices. Transfected cells were plated into 96-well plates. Then 100 μl medium containing 50 μM EdU was added into each well for incubation. After cell fixation and staining, images were captured using a fluorescence microscopy. Three repeated assays were performed.

### Colony formation assay

Cells after transfection were seeded in the six-well plates. 10 days later, visible colonies were counted under a microscope after cells went through fixation and staining. Experiments were repeated three times.

### JC-1 assay

Cells were first treated with JC-1 solution (Beyotime, China). Then, the fluorescence-labeled cells were washed and analyzed using an EnSpire Reader. Experiments were repeated three times.

### Western blot

Total protein was extracted and the concentrations were quantified. Next, the proteins were separated by SDS-PAGE, passed on to PVDF membranes (Millipore, USA). After being sealed in 5% nonfat milk, the membranes were incubated with anti-Bax (ab32503, Abcam, 1/1000), anti-Bcl-2 (ab32124, Abcam, 1/1000), anti-cleaved caspase-3 (ab2302, Abcam, 1/500), anti-E-cadherin (ab40772, Abcam, 1/10000), anti-N-cadherin (ab98952, Abcam, 1/2000), anti-ALKBH5 (ab195377, Abcam, 1/1000), anti-KCNK15 (abs147322, Absin, 1/500), anti-β-catenin (ab32572, Abcam, 1/5000), anti-p65 (ab32536, Abcam, 1/1000), anti-histone H3 (ab1791, Abcam, 1/5000), anti-p-ERK1/2 (ab214362, Abcam, 1/400), anti-ERK1/2 (ab184699, Abcam, 1/10000), anti-NOTCH1 (ab52627, Abcam, 1/1000), anti-HES1 (ab108937, Abcam, 1/500), anti-p-AKT (ab38449, Abcam, 1/500), anti-AKT (ab8805, Abcam, 1/500), anti-PTEN (ab170941, Abcam, 1/1000), anti-FOXP1 (ab32010Abcam, 1/500), anti-EZH2 (ab186006, Abcam, 1/500), anti-REST (ab21635, Abcam, 1/100), anti-MDM2 (ab226939, Abcam, 1/1000), anti-alpha Tubulin (ab7291, Abcam, 1/10000) and anti-GAPDH (ab8245, Abcam, 1/10000) primary antibodies overnight at 4 °C. Next, membranes were hatched with secondary antibody conjugated with horseradish peroxidase. The signals were measured via ECL western blotting substrate (Invitrogen, USA). Each sample was tested in triplicate.

### Wound healing assay

Cells were planted into six-well plates and allowed to reach 80% confluence. A sterilized 200 μl pipette tip was adopted for a straight scratch. Images were observed using a microscopy at the indicated times, and wound width at 24 h relative to 0 h was evaluated to assess wound healing. Experiments were repeated three times.

### Immunofluorescence (IF) staining

Cells were plated into six-well plates. Afterwards, cells were treated with primary antibodies against E-cadherin (ab40772, Abcam, 1/500) and N-cadherin (ab98952, Abcam, 1/500), followed by incubation with fluorochrome-labeled secondary antibody. Cell nuclei were stained with DAPI and then imaged via a fluorescence microscopy. Experiments were repeated three times.

### RNA binding protein immunoprecipitation (RIP) assay

The Magna RNA-binding protein immunoprecipitation kit (Millipore) was adopted for this experiment in the light of the instructions. METTL3 (ab195352, Abcam, 1/50), METTL14 (26158-1-AP, Proteintech, 1/50), WTAP (ab195380, Abcam, 1/40), FTO (#31687, Cell signaling technology, 1/50), ALKBH5 (16837-1-AP, Proteintech, 1/50), MDM2 (ab226939, Abcam, 1/100), and IgG (#3900, Cell signaling technology, 1/50) control antibodies were adopted. Finally, RNA complexes were purified and examined by RT-qPCR. Experiments were repeated three times.

### Luciferase reporter assay

For detecting the luciferase activity of gene promoter, cells were transfected with pGL3 reporter vectors (Promega, USA) inserted with KCNK15-AS1 promoter or PTEN promoter, and further co-transfected with the indicated plasmids. 48 h later, a luciferase reporter assay kit was adopted to assess promoter activity.

To test the impact of ALKBH5 on KCNK15-AS1 with or without m^6^A motifs, luciferase reporter assays were performed. In detail, KCNK15-AS1 containing wild-type (WT) or mutant (Mut) m^6^A consensus sequences was inserted into pmirGLO dual luciferase vectors, which was then transfected into PC cells. Two days later, the luciferase activities were monitored by a dual luciferase reporter assay kit.

To determine the interaction between KCNK15-AS1 and KCNK15, cells were transfected with pmirGLO dual luciferase vectors containing KCNK15 5′UTR and KCNK15 3′UTR, together with KCNK15-AS1 overexpression plasmids. After 48 h, the activity was measured via a dual luciferase reporter assay kit. Experiments were repeated three times.

### Methylated RNA immune‑precipitation (MeRIP) assay

This assay was conducted by using a MeRIP m^6^A Kit (Merck Millipore) following provider’s requirements. In detail, total RNA was first separated from cells and incubated with m^6^A antibody (202 003, Synaptic Systems) to precipitate KCNK15-AS1. Abundance of KCNK15-AS1 in precipitates was examined via RT-qPCR. The experiments were repeated three times.

### Subcellular fractionation assay

A PARIS kit (Thermo Fisher, USA) was employed for isolating the cytoplasmic and nuclear RNA following the protocols. KCNK15-AS1 expression in the cytoplasm and nucleus was analyzed by RT-qPCR. GAPDH and U6 were used for internal references. Three repeated experiments were performed.

### Fluorescent in situ hybridization (FISH)

Cells were incubated with KCNK15-AS1 probes in hybridization solution after fixation and permeabilization. Nuclei were dyed with DAPI. Images were observed via a microscope. Experiments were repeated three times.

### Chromatin immunoprecipitation (ChIP)

The EZ ChIP^TM^ Chromatin Immunoprecipitation Kit (Millipore, USA) was employed for ChIP assays conforming to the instructions. Besides, anti-REST (17-10456, Sigma-Aldrich) was adopted and IgG was control. The bound DNA was examined using qPCR. Experiments were repeated three times.

### Cycloheximide (CHX) measurements

The KCNK15-AS1 overexpression plasmids were transfected into cells, and CHX (10 ug/ml) was added for incubation for 0, 6, and 12 h. The level of REST in cell lysates were detected by western blot using anti-REST (ab21635, Abcam) antibody. Experiments were repeated three times.

### Ubiquitination assay

Ubiquitin and KCNK15-AS1 plasmids were transfected into cells. Then cell lysates were incubated with the anti-REST (22242-1-AP, Proteintech) conjugated with protein A/G beads. Following boiling and elution, protein levels were examined via western blot. Experiments were repeated three times.

### RNA pulldown assay

The Pierce™ Magnetic RNA-Protein Pull-Down (Thermo Scientific, USA) was used for this experiment. Cell extracts were mixed with biotinylated KCNK15-AS1 sense or KCNK15-AS1 antisense and streptavidin beads. After washing and boiling, the RNA complex was detected via western blot. The experiments were conducted three times.

### Co-immunoprecipitation (Co-IP)

For Co-IP experiment, anti-REST (22242-1-AP, Proteintech), anti-MDM2 (ab226939, Abcam, 1/100) or normal IgG were employed. The protein complex was examined using western blot. Three repeated experiments were carried out.

### Statistical analyses

Data from three repeated experiments were shown as mean ± standard deviation and programed via Graphad Prism 6 software. Student’s *t* test, one-way or two-way analysis of variance was appropriately applied to assess group differences. *P* < 0.05 suggested statistical significance.

## Results

### KCNK15-AS1 inhibits PC cell growth

**Fig. 1:**
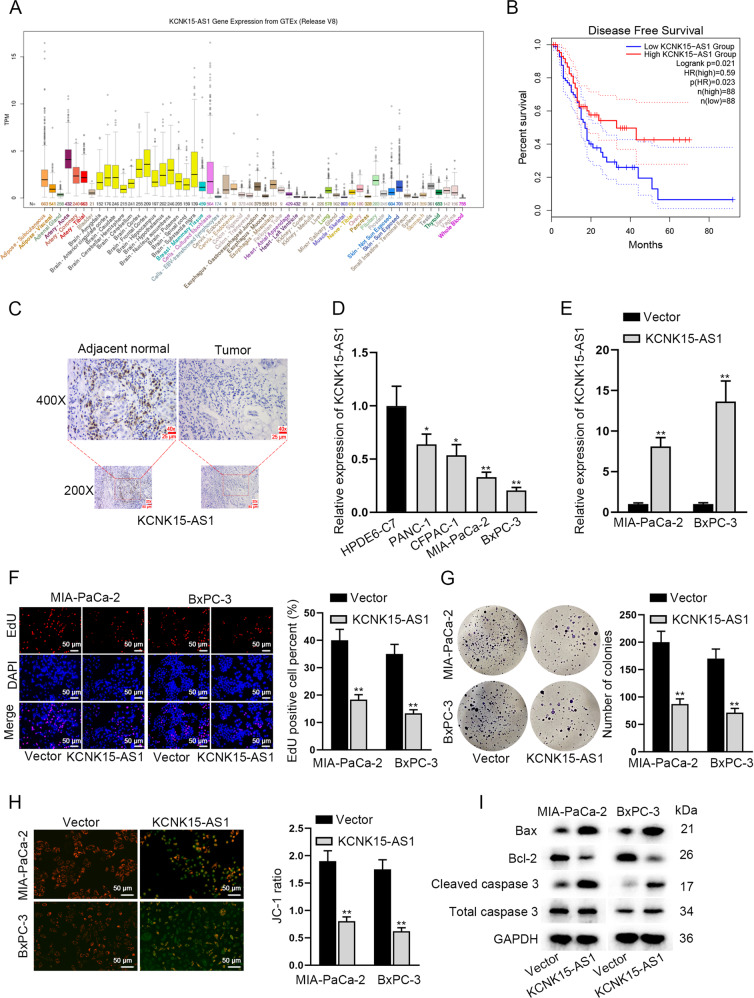
KCNK15-AS1 inhibits PC cell growth. **A** KCNK15-AS1 expression in different normal tissues was revealed by UCSC. **B** GEPIA 2 data disclosed the correlation between disease free survival (DFS) and KCNK15-AS1 expression in PC patients. **C** KCNK15-AS1 expression was determined by ISH assay in PC samples and paired adjacent tissues (scale bar: 40×, 25 μm; 20×, 50 μm). **D** KCNK15-AS1 expression in human PC cells relative to normal HPDE6-C7 cells was determined by RT-qPCR. **E** RT-qPCR analysis validated KCNK15-AS1 overexpression efficiencies in PC cells. **F**, **G** PC cell proliferation was examined by EdU (scale bar: 50 μm) and colony formation assays. **H** PC cell apoptosis was assessed by JC-1 assays. **I** Impact of KCNK15-AS1 on apoptosis-related proteins including Bax, Bcl-2 and cleaved caspase-3 was analyzed by western blot in PC cells. ^*^P < 0.05, ^**^P < 0.01.

A previous study has demonstrated that KCNK15-AS1 is downregulated in PC cells and impairs PC cell motility [13]. Here, we wondered the regulation of KCNK15-AS1 on PC cell growth. Hence, we analyzed the relevance of KCNK15-AS1 to PC. As illustrated in Fig. 1A, UCSC (http://genome.ucsc.edu) data indicated that KCNK15-AS1 level was low in normal pancreatic tissues. Significantly, data from GEPIA 2 (http://gepia2.cancer-pku.cn) unveiled that low KCNK15-AS1 expression was related to short disease free survival of PC patients (median value of KCNK15-AS1 level was regarded as cutoff value, *p* = 0.021) (Fig. 1B). Moreover, ISH assay results indicated that the expression of KCNK15-AS1 in PC samples was lower than that in adjacent normal samples (Fig. 1C). Also, RT-qPCR analysis validated that KCNK15-AS1 was low-expressed in PC cell lines compared with human normal pancreatic ductal epithelial cell line HPDE6-C7, and MIA-PaCa-2 and BxPC-3 cells exhibited the lowest KCNK15-AS1 level (Fig. 1D). On this basis, MIA-PaCa-2 and BxPC-3 cells were selected for subsequent assays. To examine the effect of KCNK15-AS1 on PC cell proliferation and apoptosis, we transfected MIA-PaCa-2 and BxPC-3 cells with pcDNA3.1/KCNK15-AS1 to induce overt upregulation of KCNK15-AS1 (Fig. 1E). Later, it manifested that KCNK15-AS1 overexpression hampered PC cell proliferation (Fig. 1F, G), while facilitated PC cell apoptosis (Fig. 1H). Meanwhile, western blot results exhibited that KCNK15-AS1 overexpression led to remarkable increase in the levels of Bax and cleaved caspase-3 whereas a decrease in Bcl-2 protein level (Fig. 1I). Taken together, KCNK15-AS1 hinders cell proliferation and enhances cell apoptosis in PC.

### KCNK15-AS1 restrains cell migration and EMT in PC

**Fig. 2:**
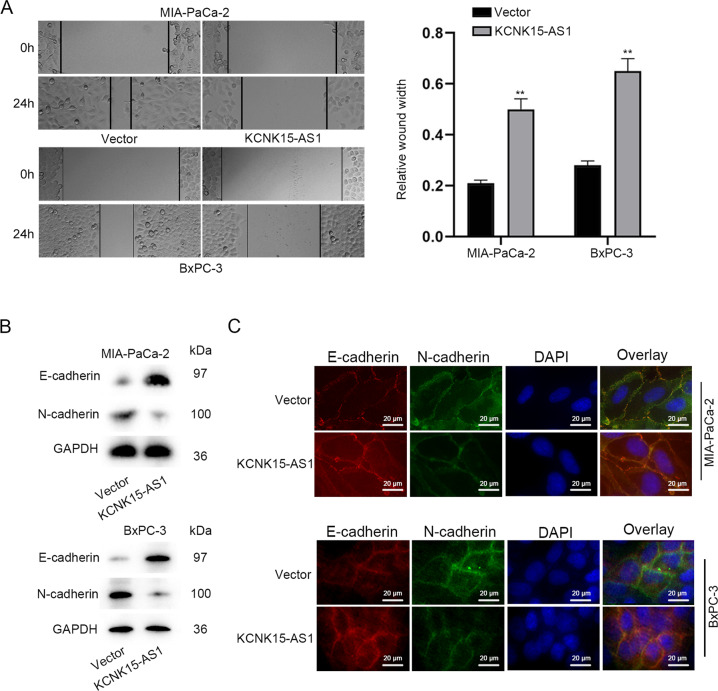
KCNK15-AS1 inhibits cell migration and EMT in PC. **A** Cell migration was monitored by wound healing assay. **B**, **C** The influence of KCNK15-AS1 on EMT markers (E-cadherin and N-cadherin) in PC cells was measured using western blot and immunofluorescence staining (scale bar: 20 μm). ^**^P < 0.01.

Additionally, the impacts of KCNK15-AS1 on PC cell migration and EMT were also evaluated. Based on the results of wound healing assays, we confirmed that overexpressing KCNK15-AS1 obviously weakened PC cell migration (Fig. 2A). Consistently, elevation of KCNK15-AS1 resulted in increased expression of epithelial marker (E-cadherin) and lowered level of mesenchymal marker (N-cadherin) (Fig. 2B, C), indicating that KCNK15-AS1 impeded EMT process of PC cells. To conclude, KCNK15-AS1 inhibits PC cell migration and EMT.

### ALKBH5 induces m^6^A demethylation to mediate KCNK15-AS1 upregulation in PC cells

**Fig. 3:**
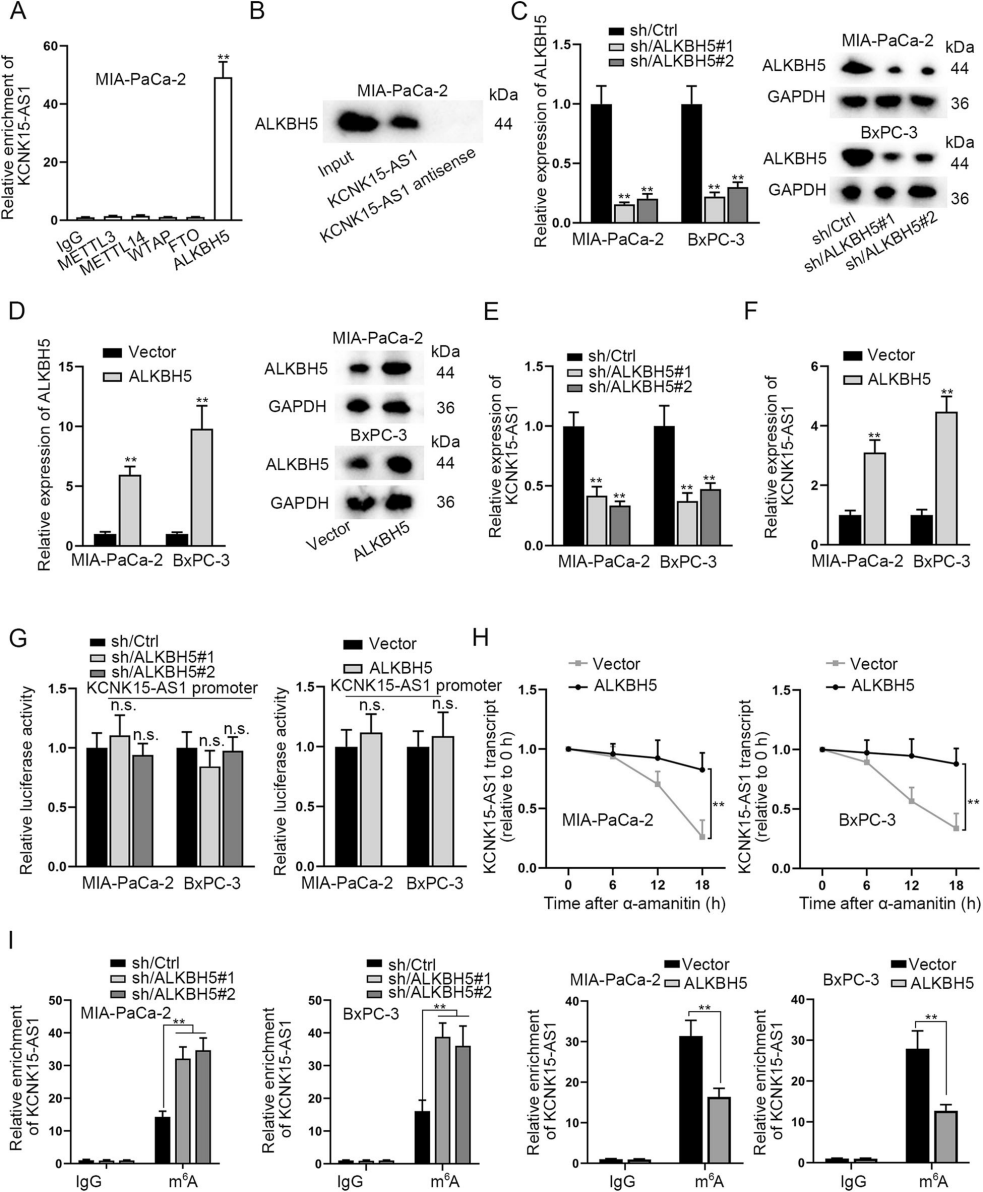
ALKBH5 binds with and stabilizes KCNK15-AS1 through m^6^A-dependent manner. **A** Interaction of KCNK15-AS1 with METTL3, METTL14, WTAP, FTO and ALKBH5 in PC cells was measured by RIP assay (IgG served as negative control). **B** RNA pulldown assay was performed to evaluate the binding of ALKBH5 with KCNK15-AS1 in PC cells. **C**, **D** Knockdown and overexpression efficiencies of ALKBH5 were tested by RT-qPCR and western blot in PC cells. **E**, **F** KCNK15-AS1 expression in PC cells after ALKBH5 silence or overexpression was examined using RT-qPCR. **G** Luciferase activity of KCNK15-AS1 promoter was measured in PC cells with ALKBH5 silence or overexpression. **H** The level of KCNK15-AS1 in indicated PC cells under α-amanitin (50 mM) treatment was measured by RT-qPCR. **I** RIP assay was carried out to detect the impact of ALKBH5 on m^6^A modification in KCNK15-AS1 in PC cells. ^**^P < 0.01, n.s. no significance.

We then probed the possible mechanism of KCNK15-AS1 in PC cells. Since m^6^A modification has been well-documented to affect lncRNA expression, we wondered whether KCNK15-AS1 was modulated via m^6^A-dependent manner. According to the data of RIP assays, we found that only antibodies against ALKBH5 obviously precipitated KCNK15-AS1 (Fig. 3A), indicating the strong binding affinity between KCNK15-AS1 and ALKBH5 in PC cells. Likewise, the outcome of RNA pulldown experiment confirmed the binding between KCNK15-AS1 and ALKBH5 (Fig. 3B). Furthermore, we discovered that ALKBH5 knockdown led to a remarkable reduction in KCNK15-AS1 expression, whereas it up-regulation resulted in opposite phenomena (Fig. 3C–F). Intriguingly, we found that alterations in ALKBH5 had no apparent effect on the luciferase activity of KCNK15-AS1 promoter (Fig. 3G), which suggested that ALKBH5 could not affect KCNK15-AS1 transcription. Moreover, we uncovered that elevation of ALKBH5 prolonged the half-life of KCNK15-AS1 in two PC cell lines (Fig. 3H). Next, we assessed the influence of ALKBH5 on m^6^A level in KCNK15-AS1 via MeRIP assays. As a result, we discovered that ALKBH5 knockdown or overexpression increased or decreased the enrichment of KCNK15-AS1 in anti-m^6^A group, respectively (Fig. 3I), validating that ALKBH5 negatively regulated m^6^A modification on KCNK15-AS1 in PC cells. It has been accepted that m^6^A modification occurs at RRACH motifs (R = G or A; H = A, C or U). As shown in Additional file 1, we found 11 m^6^A consensus sequences in KCNK15-AS1. Then, we mutated these sites (5′-RRACH-3′ to 5′-RRUCH-3′) to abolish m^6^A modifications, and subsequently conducted luciferase reporter assays. Results manifested that ALKBH5 had positive modulation on the luciferase activity of KCNK15-AS1-WT while had no impact on KCNK15-AS1-Mut activity (Figs. S1A, S1B). In summary, ALKBH5 positively modulates KCNK15-AS1 in PC cells through its m^6^A demethylation ability.

### KCNK15-AS1 inhibits KCNK15 translation via binding to KCNK15 5’UTR

**Fig. 4:**
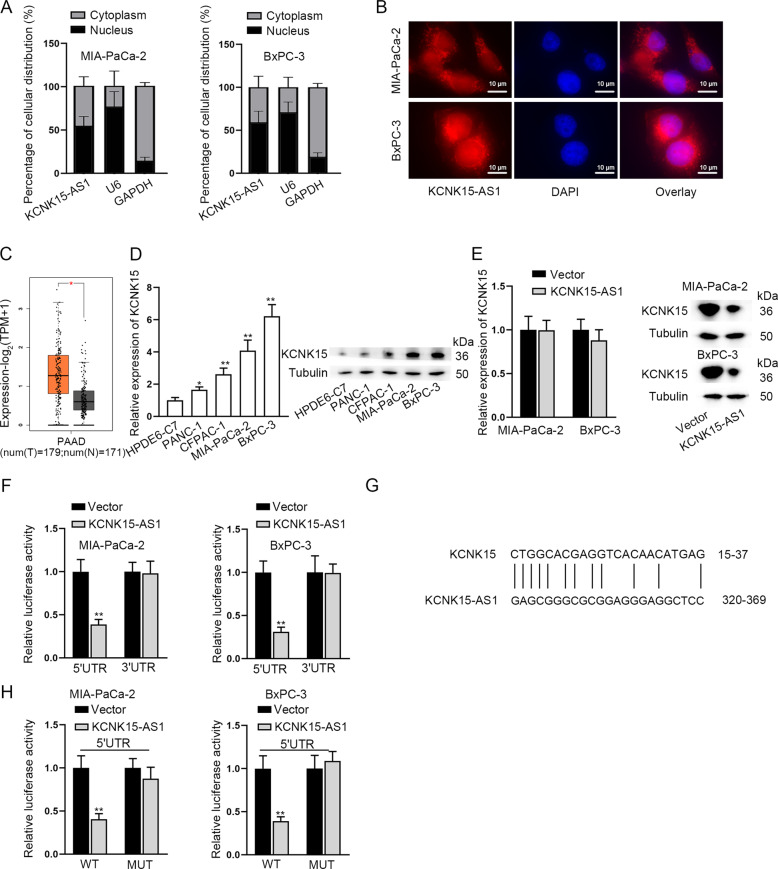
KCNK15-AS1 represses KCNK15 translation via binding to KCNK15 5’UTR. **A**, **B** Subcellular fractionation and FISH assays (Scale bar: 10 μm) were performed to identify the cellular location of KCNK15-AS1 in PC cells. **C** Data of KCNK15 expression in PAAD tissues (N = 179) and normal tissues (*N* = 171) was obtained from GEPIA 2. **D** KCNK15 expression in human PC cells relative to HPDE6-C7 cells was determined by RT-qPCR and western blot. **E** RT-qPCR and western blot analyzed the influence of KCNK15-AS1 on KCNK15 expression in PC cells. **F** Luciferase activities of KCNK15 5’UTR and KCNK15 3′UTR were detected in PC cells with KCNK15-AS1 overexpression. **G** Binding sequences between KCNK15-AS1 and KCNK15 5’UTR was shown. H Luciferase activities of KCNK15 5′UTR-WT (with wild-type binding sites) and KCNK15 5′UTR-MUT (with mutated sites) were detected in PC cells after KCNK15-AS1 overexpression. ^*^P < 0.05, ^**^P < 0.01.

Then, we investigated the downstream mechanism of KCNK15-AS1 in PC cells. Before that, we proved that KCNK15-AS1 was located in both the nucleus and cytoplasm of PC cells (Fig. 4A, B). For all we know, lncRNAs exert their functions via cooperating with their nearby genes [25]. Herein, we suspected that KCNK15-AS1 might affect the expression of its nearby gene KCNK15 in PC. Based on GEPIA 2 data, we found that KCNK15 level was higher in PAAD tissues in comparison with normal tissues (Fig. 4C**, num (T)** = **179; num (N)** = **171**). Furthermore, KCNK15 levels were revealed to be up-regulated in PC cell lines relative to HPDE6-C7 cells (Fig. 4D). More importantly, we unmasked that KCNK15-AS1 up-regulation could not influence KCNK15 mRNA level, but decreased the protein level of KCNK15 (Fig. 4E). Luciferase reporter assays further confirmed that overexpressed KCNK15-AS1 significantly reduced the activity of KCNK15 5′UTR and barely affected that of KCNK15 3′UTR (Fig. 4F). Of note, by comparing the sequences of KCNK15 5’UTR and KCNK15-AS1, we found matching bases between them (Fig. 4G). Thus, we speculated that KCNK15-AS1 directly bound to KCNK15 5′UTR to affect KCNK15 translation. To validate the speculation, we conducted luciferase reporter assay and found that the luciferase activity of KCNK15 5′UTR-WT but not KCNK15 5′UTR-MUT was obviously lessened by KCNK15-AS1 overexpression in PC cells (Fig. 4H). Collectively, KCNK15-AS1 represses KCNK15 translation via directly binding to KCNK15 5′UTR.

### KCNK15-AS1 partly depends on KCNK15 to suppress malignant phenotypes of PC cells

Further, we carried out rescue experiments to investigate the effect of KCNK15-AS1/KCNK15 axis on PC cellular functions. First, we verified KCNK15 overexpression in PC cells after pcDNA3.1/KCNK15 transfection (Fig. S2A). It showed that KCNK15 upregulation partly recovered the effect of KCNK15-AS1 overexpression on the proliferation and apoptosis of PC cells (Fig. S2B–S2E). Moreover, cell migration inhibited by KCNK15-AS1 upregulation was partially reversed after KCNK15 overexpression in PC cells (Fig. S2F). Additionally, KCNK15 overexpression partly offset the changes in E-cadherin and N-cadherin caused by KCNK15-AS1 overexpression in PC cells (Fig. S2G). To sum up, KCNK15-AS1 restrains PC cell malignant behaviors partly via repressing KCNK15.

### KCNK15-AS1 suppresses REST to inactivate PTEN/AKT pathway

**Fig. 5:**
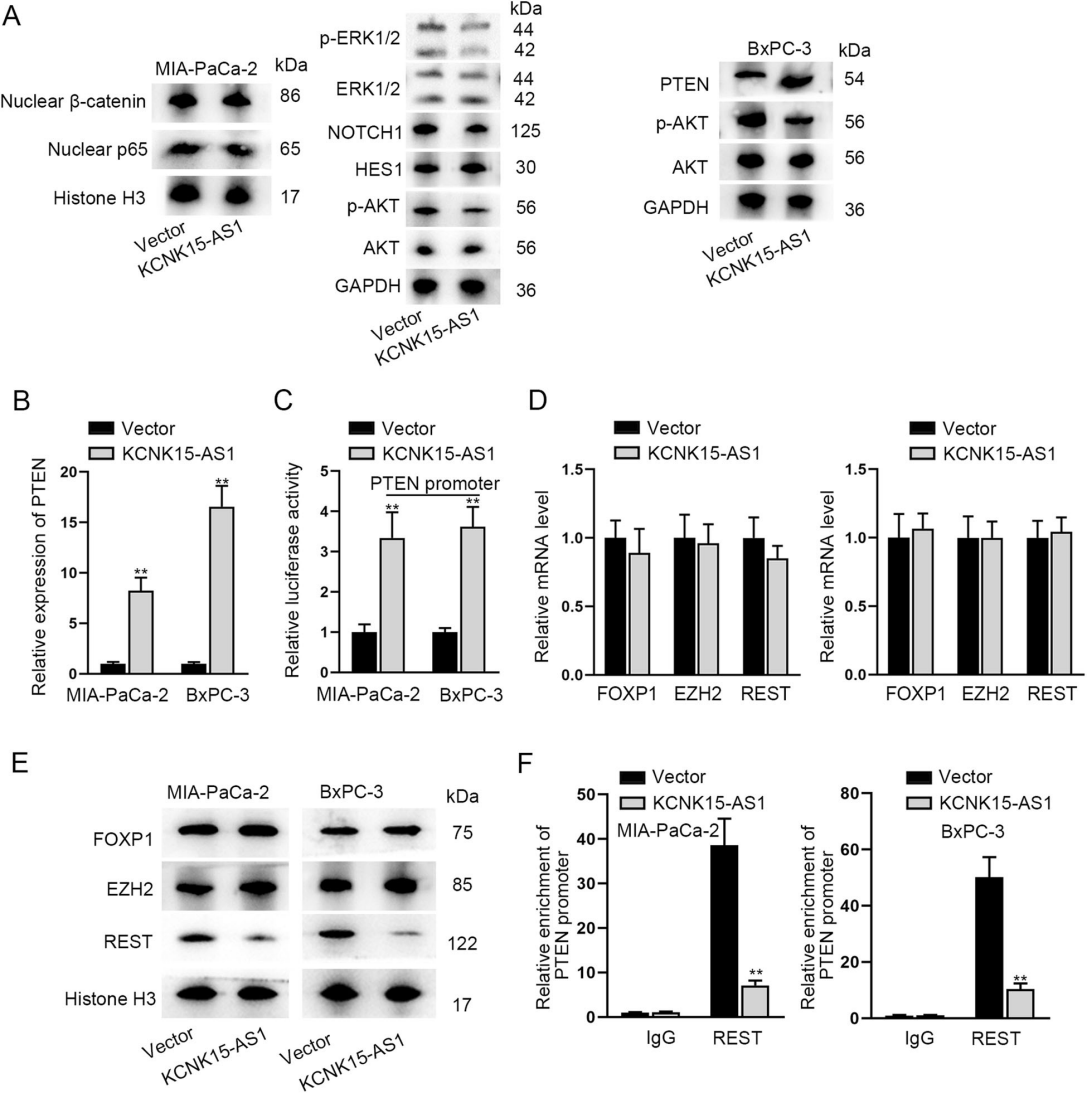
KCNK15-AS1 inhibits REST to promote PTEN transcription and inactivate AKT pathway in PC cells. **A** Levels of key proteins in several signaling pathways were analyzed by western blot in PC cells after KCNK15-AS1 overexpression. **B** PTEN expression was analyzed using RT-qPCR in PC cells after KCNK15-AS1 overexpression. **C** Luciferase activity of PTEN promoter was detected in PC cells after KCNK15-AS1 overexpression. **D**, **E** Influence of KCNK15-AS1 on FOXP1, EZH2 and REST expression was detected using RT-qPCR and western blot. **F** Impact of KCNK15-AS1 on the binding of REST to PTEN promoter was measured via ChIP assays. ^**^P < 0.01.

Since KCNK15-AS1 functioned in PC partly through KCNK15, we further investigated other potential ways. It is well known that dysregulation of signaling pathways is important for cancer development. Therefore, we examined the influences of KCNK15-AS1 on the levels of key proteins in several well-characterized cancer-related signaling pathways including Wnt/β-catenin pathway, NF-kappaB pathway, ERK pathway, NOTCH pathway, and AKT pathway. As depicted in Fig. 5A, KCNK15-AS1 overexpression markedly reduced p-AKT level, whereas had no effect on other proteins. Furthermore, we found that along with the reduced level of p-AKT, the protein level of AKT inactivator PTEN was synchronously enhanced in KCNK15-AS1 upregulated PC cells. Further, it manifested that KCNK15-AS1 overexpression markedly enhanced the mRNA level of PTEN in two PC cell lines (Fig. 5B). Meanwhile, we found that elevated expression of KCNK15-AS1 dramatically enhanced the luciferase activity of PTEN promoter (Fig. 5C), suggesting that KCNK15-AS1 accelerated PTEN transcription. Browsing UCSC database, we found three well-known transcription factors (FOXP1, EZH2, and REST) potentially binding to PTEN promoter. Data presented that KCNK15-AS1 overexpression could not influence the mRNA levels of all three transcription factors, but reduced the protein level of nuclear REST (Fig. 5D, E). ChIP assay results validated that the interaction between REST and PTEN promoter was hindered by KCNK15-AS1 (Fig. 5F). Through JASPAR (http://jaspar.genereg.net), we found three REST binding sites in PTEN promoter, and then fragmented PTEN promoter into two segments (P1 and P2) (Fig. S3A–S3B). The results of ChIP assays further verified that P2 part of PTEN promoter was recognized by REST (Fig. S3C). We additionally found that PTEN was downregulated by REST overexpression (Fig. S3D). Moreover, luciferase reporter assay results validated that after REST overexpression, the luciferase activity of PTEN promoter-WT was overtly inhibited, that of PTEN promoter with S1-MUT or S2-MUT was moderately lessened, while that of PTEN promoter with S1 + S2-MUT was almost not altered in PC cells (Fig. S3E). Taken together, KCNK15-AS1 reduces REST protein to facilitate PTEN transcription and therefore inhibit AKT pathway in PC cells.

### KCNK15-AS1 facilitates REST ubiquitination via recruiting MDM2

**Fig. 6:**
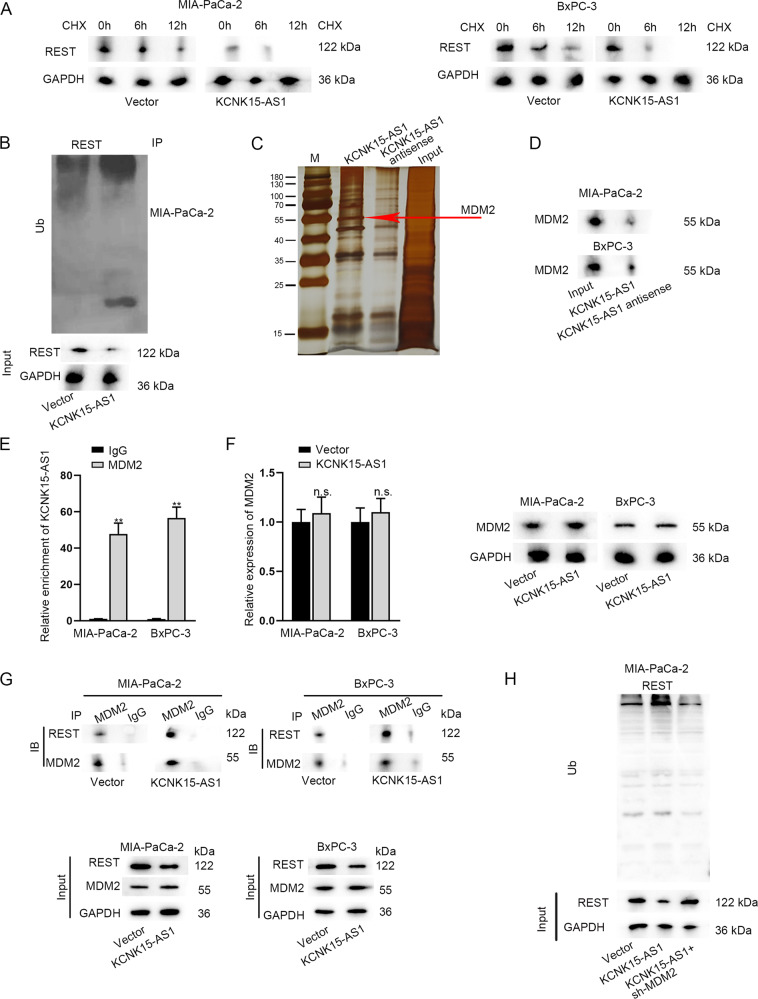
KCNK15-AS1 recruits MDM2 to promote REST ubiquitination. **A** Influence of KCNK15-AS1 on REST degradation under CHX treatment was detected using western blot. **B** REST ubiquitination was analyzed by IP analysis in MIA-PaCa-2 cells with or without KCNK15-AS1 overexpression. **C** RNA pulldown and mass spectrometry were performed to analyze KCNK15-AS1-interacting proteins in PC cells. **D**, **E** Interaction between KCNK15-AS1 and MDM2 protein was confirmed by RNA pulldown and RIP assays. **F** Effect of KCNK15-AS1 augment on MDM2 expression in PC cells was measured using RT-qPCR and western blot. **G** Co-IP assay was conducted to detect the impact of KCNK15-AS1 on the interaction between REST and MDM2 in PC cells. **H** IP analysis was done to test REST ubiquitination levels in indicated PC cells. ^**^P < 0.01, n.s. no significance.

We further probed into the way how KCNK15-AS1 regulated REST protein in PC cells. It manifested that the decrease of REST protein under CHX treatment was accelerated under KCNK15-AS1 overexpression (Fig. 6A), indicating that KCNK15-AS1 decreased REST protein stability in PC cells. Moreover, the ubiquitination level of REST was increased by overexpressed KCNK15-AS1 in PC cells (Fig. 6B). Then, RNA pulldown experiment and mass spectrometry were conducted. It turned out that KCNK15-AS1 might interact with MDM2, a specific protein at about 55 kDa (Fig. 6C). Further, the binding of MDM2 with KCNK15-AS1 in PC cells was confirmed by RNA pulldown and RIP assays (Fig. 6D, E). MDM2 is an E3 ubiquitin ligase which promotes the ubiquitination of target proteins [26]. Therefore, we assumed that KCNK15-AS1 might promote REST ubiquitination through MDM2-mediated way. However, we found that KCNK15-AS1 up-regulation could not affect MDM2 expression (Fig. 6F), but strengthened the interaction between MDM2 and REST (Fig. 6G). Moreover, we confirmed that KCNK15-AS1 overexpression increased REST ubiquitination level while such effect was counteracted by MDM2 knockdown (Fig. 6H). These data certified that KCNK15-AS1 promotes REST ubiquitination via interacting with MDM2.

### KCNK15-AS1 regulates KCNK15 and PTEN to induce suppression on PC cells

Thereafter, rescue assays were performed to determine whether KCNK15-AS1 repressed PC cell functions via regulating both KCNK15 and PTEN. Before that, we interfered PTEN in PC cells (Fig. S4A). Results of EdU and colony formation assays validated that cell proliferation restricted by KCNK15-AS1 overexpression was partly restored by PTEN interference, and was fully restored in KCNK15-AS1 + KCNK15 + sh/PTEN#1 group (Fig. S4B–S4C). Moreover, KCNK15-AS1 overexpression-enhanced cell apoptosis was partially reversed by PTEN knockdown, and was fully reversed under PTEN inhibition and KCNK15 overexpression (Fig. S4D–S4E). Moreover, PTEN deletion moderately neutralized the inhibition on cell migration and EMT caused by overexpressed KCNK15-AS1, while such inhibitory effects were completely offset by PTEN depletion and KCNK15 overexpression (Fig. S4F–S4G). In sum, KCNK15-AS1 represses PC cell malignant behaviors via regulating both KCNK15 and PTEN.

## Discussion

Recent evidence has proved the importance of lncRNAs in PC progression. KCNK15-AS1 has been reported to suppress lung cancer cell proliferation [27]. Notably, KCNK15-AS1 was reported to be low-expressed in PC tissues and its upregulation repressed PC cell migration and invasion [13]. In current research, we also confirmed that KCNK15-AS1 expression was low in PC cells, and additionally discovered that low KCNK15-AS1 expression predicted poor survival of PC patients. Moreover, KCNK15-AS1 restrained PC cell proliferation, migration, and EMT. These results supported that KCNK15-AS1 acted as a tumor suppressor in PC cells.

Our study also discovered that KCNK15-AS1 was regulated by ALKBH5 through m^6^A-dependent way. Current evidence has demonstrated that m^6^A methylation is an important post-transcriptional RNA modification [28]. ALKBH5 is considered as the demethyltransferase responsible for m^6^A demethylation [29]. As reported previously, ALKBH5 depends on its m^6^A demethylation ability to facilitate gastric cancer metastasis [30] and osteosarcoma cell proliferation [31]. Consistently, we observed that ALKBH5 blocked m^6^A modification on KCNK15-AS1 to enhance KCNK15-AS1 expression and stability in PC cells.

Furthermore, our research suggested that KCNK15-AS1 hampered the translation of its nearby gene KCNK15 via binding to KCNK15 5′UTR. KCNK15 has been regarded as a potential diagnostic and prognostic biomarker of hepatocellular carcinoma [32]. Herein, we found that KCNK15 exhibited a high level in PC cells, and it was negatively regulated by KCNK15-AS1 in PC cells. Further, rescue assays validated that KCNK15 could partially offset the suppressive effect of KCNK15-AS1 on PC cells.

Meanwhile, our study also revealed that KCNK15-AS1 increased PTEN expression to inhibit AKT pathway, a master intracellular pathway affecting multiple transcription factors and signaling molecules, such as mTOR [33]. As a negative modulator of AKT [34], PTEN is verified as a typical tumor suppressor in many cancers, including PC [24]. Former studies have indicated that PTEN can influence chemoresistance, and stemness of PC cells [35].

In depth, current study further discovered that KCNK15-AS1 modulated REST to transcriptionally promote PTEN expression. REST is a transcriptional repressor of target genes [36], and therefore serves as a promoter of tumors including PC [37]. Here, we confirmed that REST bound to PTEN promoter to reduce PTEN transcription. Furthermore, our study revealed that KCNK15-AS1 interacted with MDM2 to promote REST ubiquitination. A previous literature has clarified that MDM2 activates AKT pathway via interacting with REST and interfering with its ability to localize on p85 promoter [38]. However, our study first discovered that KCNK15-AS1 recruited MDM2 to promote REST ubiquitination, and therefore reversed the repression of REST on PTEN transcription, resulting in inactivation of downstream AKT pathway in PC cells. Significantly, rescue assay results unveiled that KCNK15-AS1 exerted tumor suppressive functions in PC cells via regulating KCNK15 and PTEN.

**Fig. 7:**
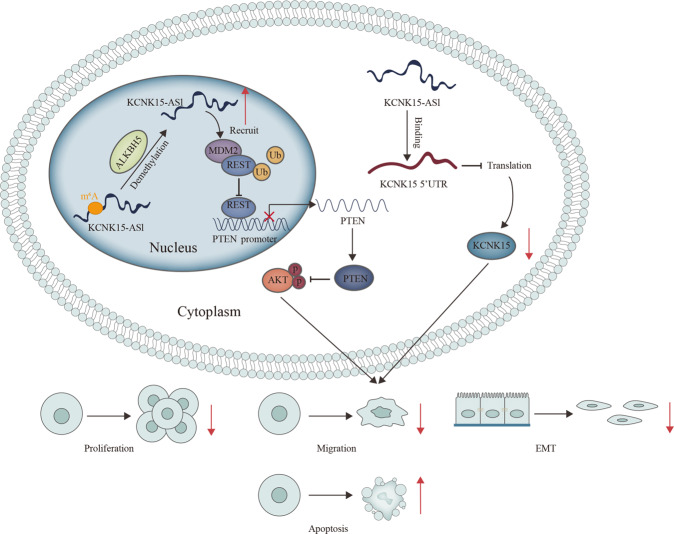
A graphical abstract was plotted to illustrate the function and mechanism of KCNK15-AS1 in pancreatic cancer. Graphical abstract illustrates the up- and down-stream mechanisms of KCNK15-AS1 in PC cells.

In conclusion, our study first demonstrated that KCNK15-AS1 suppressed PC cell proliferation, migration, and EMT. As to its mechanism, we demonstrated that ALKBH5-mediated m^6^A demethylation enhanced the stability of KCNK15-AS1. Importantly, we explained that KCNK15-AS1 bound to KCNK15 to inhibit its translation, and interacted with MDM2 to induce REST ubiquitination, which eventually facilitated PTEN transcription to inactivate AKT pathway (Fig. 7). Current data indicate that KCNK15-AS1 might serve as a valuable potential marker for PC treatment. However, we still need to make efforts to probe the potential clinical importance and in vivo function of KCNK15-AS1 in PC.

## Supplementary information

Supplemental files

Figure S1

Figure S2

Figure S3

Figure S4

Additional file 1

aj-checklist
