# Supplementary material for: ALKBH5-mediated m6A demethylation of KCNK15-AS1 inhibits pancreatic cancer progression via regulating KCNK15 and PTEN/AKT signaling
Source: Cell Death Dis. 2021 Dec 1;12(12):1121. doi: 10.1038/s41419-021-04401-4 (PMC8636648; doi:10.1038/s41419-021-04401-4)
Supplement: Supplementary file 1 — Supplemental files [file 41419_2021_4401_MOESM1_ESM.docx]

**Figure S1 ALKBH5 regulates KCNK15-AS1 through m^6^A-dependent manner.** A-B Luciferase reporter assay detected the impact of ALKBH5 on the luciferase activity of KCNK15-AS1 containing wild-type or mutant m^6^A motifs. ^*^P<0.05, ^**^P<0.01.

**Figure S2 KCNK15-AS1 inhibits PC cell malignant behaviors via down-regulating KCNK15.** A RT-qPCR was implemented to analyze KCNK15 overexpression efficiencies in PC cells. Functional assays were done when KCNK15-AS1 was overexpressed or KCNK15-AS1 and KCNK15 were co-overexpressed in PC cells. B-C Cell proliferation was detected by EdU and colony formation assays under different conditions. D Cell apoptosis was detected by JC-1 assays in cells with different transfection. E The levels of apoptosis-related proteins including Bax, Bcl-2, and cleaved caspase-3 in cells with indicated treatments were measured by western blot. F Cell migration was assessed by wound healing assays under different conditions. G The protein levels of E-cadherin and N-cadherin in cells transfected with different plasmids were measured using western blot. ^**^P<0.01.

**Figure S3 REST binds to PTEN promoter to transcriptionally inhibit PTEN.** A REST binding motif predicted by JASPAR was displayed. B Three REST binding sites in PTEN promoter predicted by JASPAR were presented. C The binding of REST to P1 or P2 part of PTEN promoter was analyzed by ChIP assays. D RT-qPCR was utilized to analyze REST (left) and PTEN (right) expressions in PC cells with or without REST overexpression. E The influence of REST overexpression on the luciferase activities of indicated PTEN promoter was examined by a luciferase reporter assay kit. ^*^P<0.05, ^**^P<0.01.

**Figure S4 KCNK15-AS1 exerts tumor-suppressive functions in PC cells via regulating KCNK15 and PTEN.** A RT-qPCR was performed to examine PTEN expression in PC cells after transfection with sh/Ctrl, sh/PTEN#1, or sh/PTEN#2. PC cell malignant processes were assessed in different groups (Vector, KCNK15-AS1, KCNK15-AS1+sh/PTEN#1, and KCNK15-AS1+KCNK15+sh/PTEN#1). B-C Cell proliferation was detected by EdU and colony formation assays under different conditions. D Cell apoptosis in different groups was detected by JC-1 assays. E The levels of apoptosis-related proteins including Bax, Bcl-2, and cleaved caspase-3 in cells transfected with indicated plasmids were tested by western blot. F Cell migration was detected by wound healing assays under different conditions. G The protein levels of E-cadherin and N-cadherin in cells with different transfection were measured using western blot. ^*^P<0.05, ^**^P<0.01.

**Additional file 1 m^6^A modification sequences in KCNK15-AS1 were displayed.** The m6A motifs were presented as sequences highlighted in yellow, and m6A modification sites were displayed as bases highlighted in red.
