# Supplementary figures and images for: ALKBH5-mediated m6A demethylation of KCNK15-AS1 inhibits pancreatic cancer progression via regulating KCNK15 and PTEN/AKT signaling
Source: Cell Death Dis. 2021 Dec 1;12(12):1121. doi: 10.1038/s41419-021-04401-4 (PMC8636648; doi:10.1038/s41419-021-04401-4)

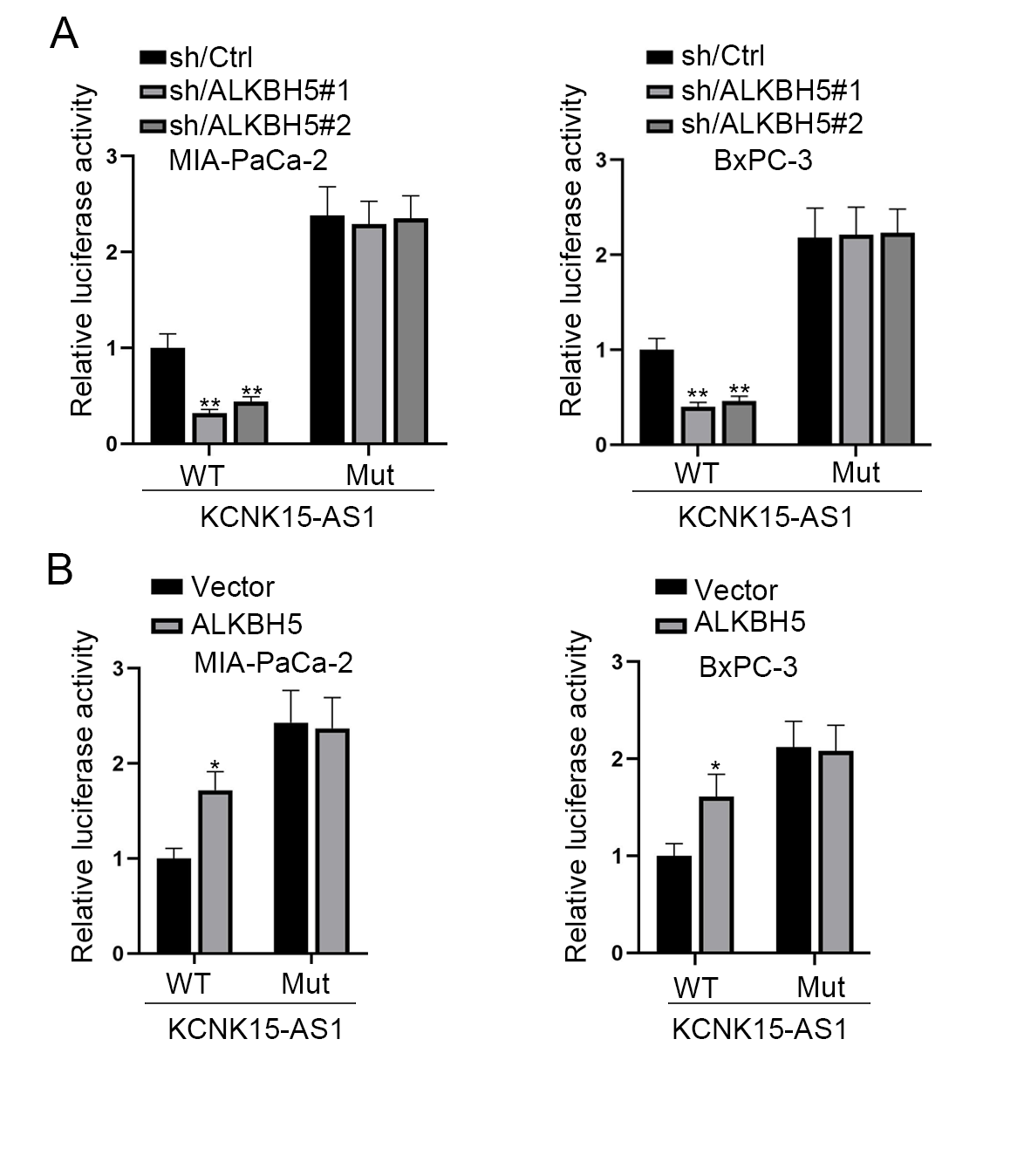

Supplement: Supplementary file 2 — Figure S1 [file 41419_2021_4401_MOESM2_ESM.tif]

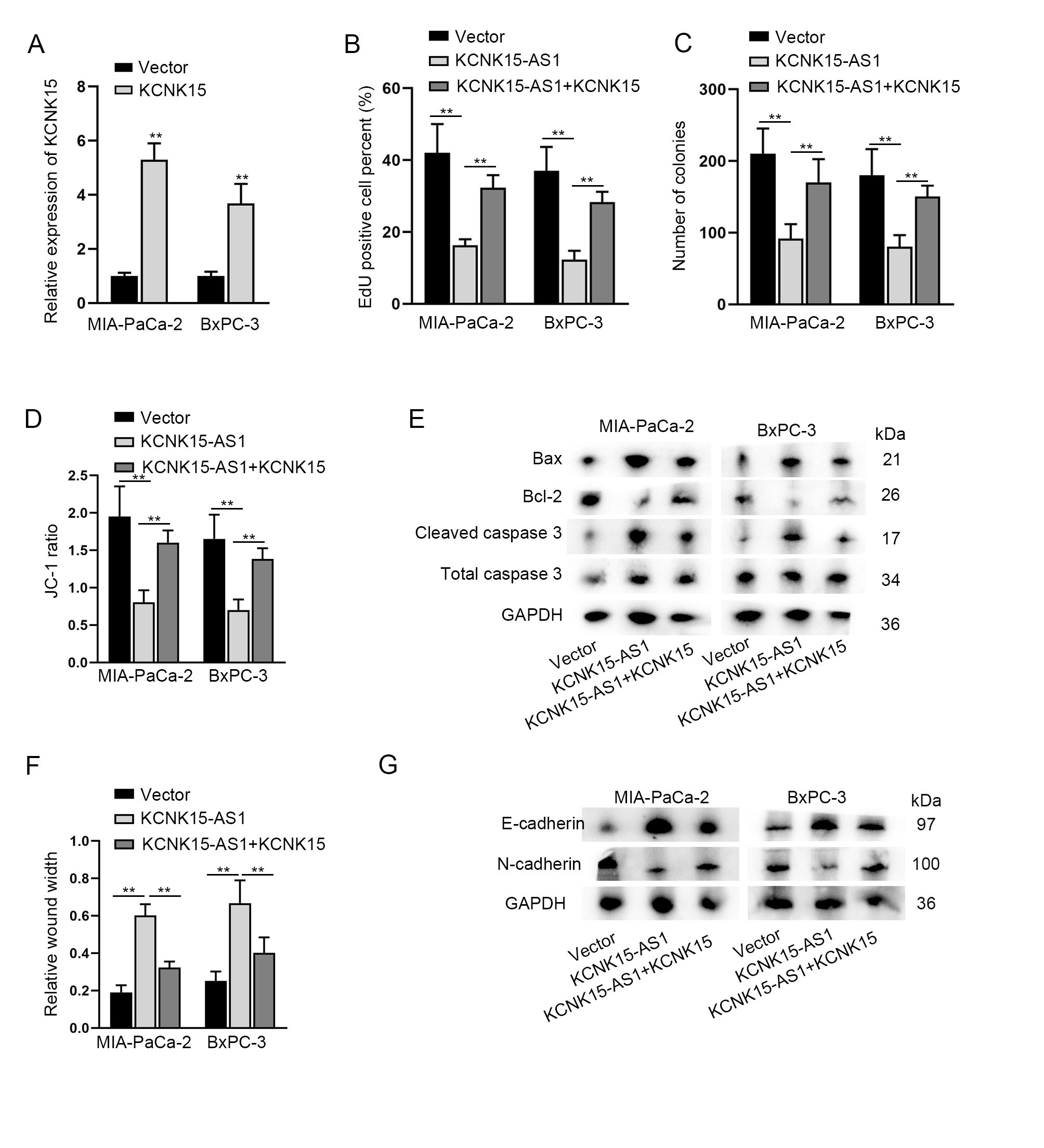

Supplement: Supplementary file 3 — Figure S2 [file 41419_2021_4401_MOESM3_ESM.tif]

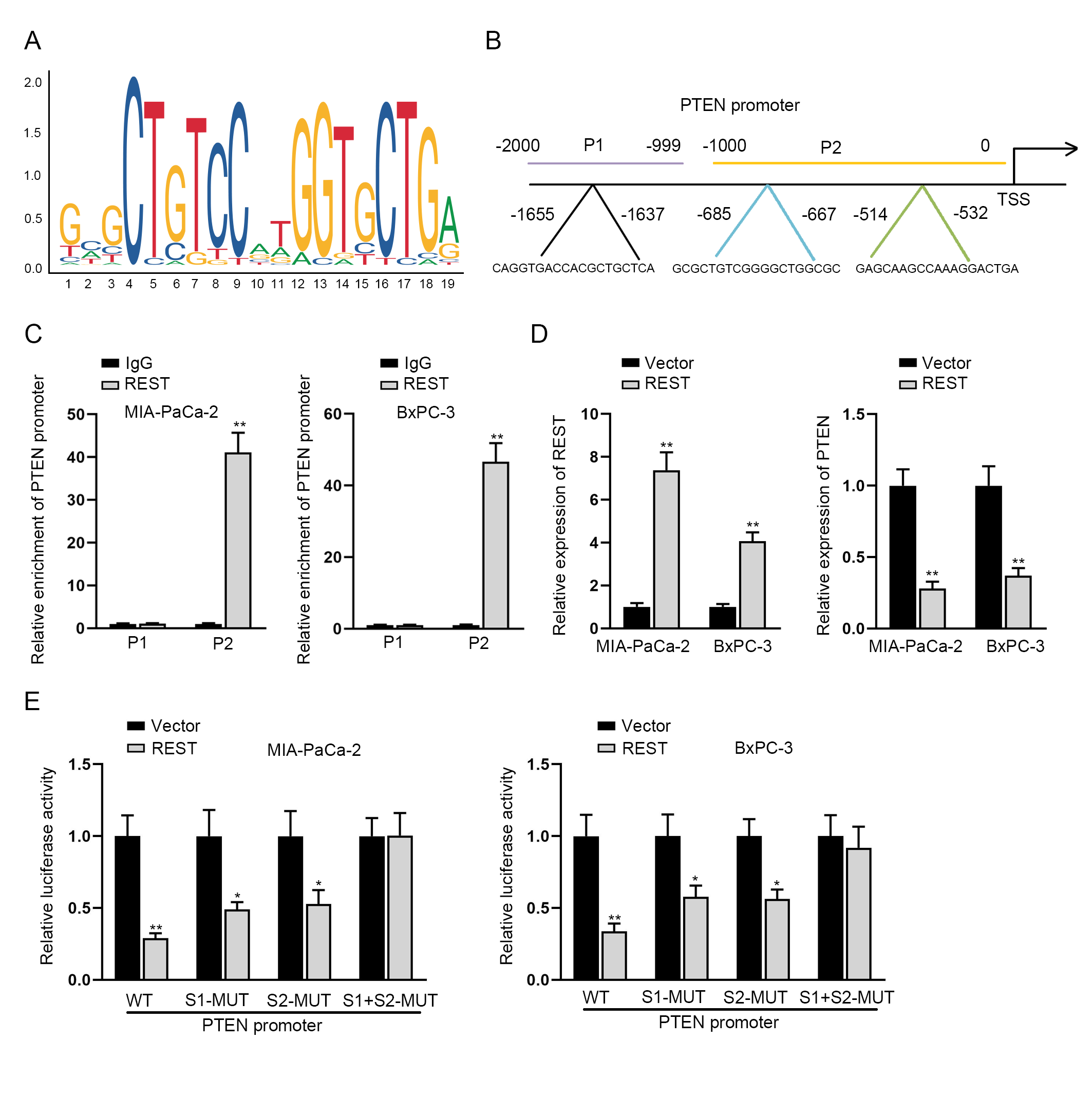

Supplement: Supplementary file 4 — Figure S3 [file 41419_2021_4401_MOESM4_ESM.tif]

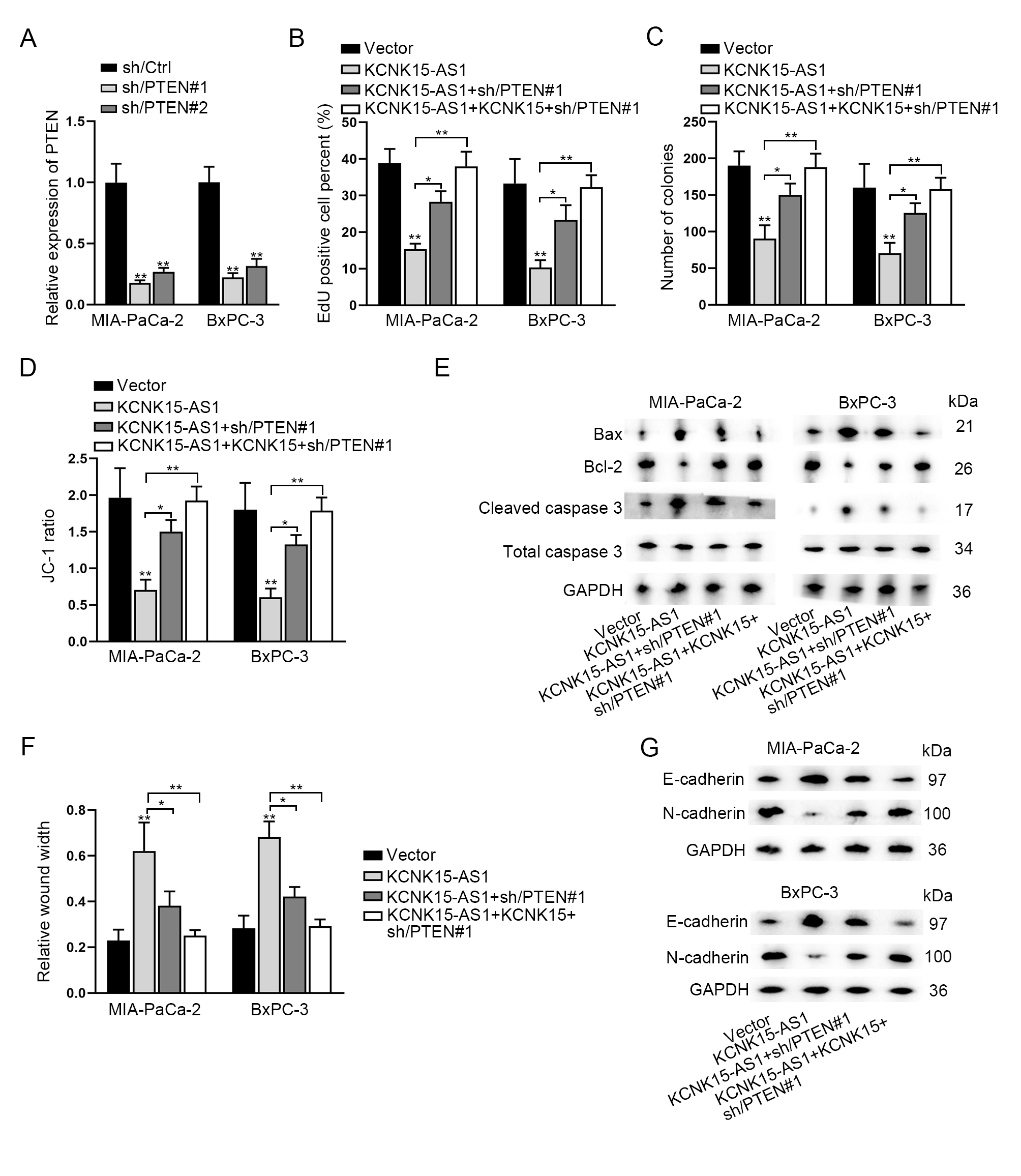

Supplement: Supplementary file 5 — Figure S4 [file 41419_2021_4401_MOESM5_ESM.tif]
