# Supplementary material for: ALKBH5-mediated m6A demethylation of KCNK15-AS1 inhibits pancreatic cancer progression via regulating KCNK15 and PTEN/AKT signaling
Source: Cell Death Dis. 2021 Dec 1;12(12):1121. doi: 10.1038/s41419-021-04401-4 (PMC8636648; doi:10.1038/s41419-021-04401-4)
Supplement: Supplementary file 6 — Additional file 1 [file 41419_2021_4401_MOESM6_ESM.docx]

>NR_132377.1 Homo sapiens KCNK15 and WISP2 antisense RNA 1 (KCNK15-AS1), long non-coding RNA

GAGCGGGCGCGGAGGGAGGCUCCGGGCGGCUCACCGAUGGUAGUGAUGACGGUGAUGGCGAAGUAGAAGGAGCCGGGGAACUUCCACUGGCGGCCGGCGCGGUGGGGCUCAGCCUGGAGCGCCAGGCGCUCCAGCUCGCGGUAGUCCUCGGCCGAGAAGCCGAACUUCCUCCGGAGAGCGCCCCGCUUCUGGACCAGCAGUCGCUGGCGGCCGCUUUCCGCCUCGGACUCGAGCGCGUCGAAGACAGCAGCGCCCACCAGCAGAUGCAGAGAACCCAAAGCCCUGCAGGAUGGCAAAGCUGCAACAUGGAAGUCACCCGGAUCACCACGUAAAGGAAAUCUCUCCAACCAGGAACAAACACCUGCCUUGCAAUAUUAGGUCCUUCCUAGGGCUUGGUGCAUGUGCAUAUCUCCUUCCGGAAUCCUGGUCAAUACUAAGGUGUCUGCCUCCUGCCCCGGUCCACAGCCAGGAGCCCCGGAGCCAACGGCCAGCCUGGAAGACUUACAGUUCUCCCUCUGAGGGUUGCUGUGGCUUUCCAUGUUGUGGUGACUAAGUCCCUGUGUUUCUGUGCAGUCCCUGCUUCCUGGGACCAGGGAAGAAUGGUCCCGUACCCGCUAUUCUGACCUCUGUGGCCACUGAUUAGUUGUUCCUGAAAGGAACAGUUUUAAGCCACUUCUAUCUCUGAGGCAAGAAGGAAGACGGGAGAAAUGAGGAGGUGUGAGGAAGAGAGUUGG

The m^6^A motifs were presented as sequences highlighted in yellow, and m^6^A modification sites were displayed as bases highlighted in red.
